# Novel *SLFN14* mutation associated with macrothrombocytopenia in a patient with severe haemorrhagic syndrome

**DOI:** 10.1186/s13023-023-02675-9

**Published:** 2023-04-11

**Authors:** Dmitrii Polokhov, Daria Fedorova, Anastasiya Ignatova, Evgeniya Ponomarenko, Elena Rashevskaya, Alexey Martyanov, Nadezhda Podoplelova, Maxim Aleksenko, Irina Mersiyanova, Elena Seregina, Aleksandr Poletaev, Ekaterina Truchina, Elena Raykina, Svetlana Plyasunova, Galina Novichkova, Pavel Zharkov, Mikhail Panteleev

**Affiliations:** 1 Dmitriy Rogachev National Research and Clinical Centre of Pediatric Hematology, Oncology and Immunology, Moscow, Russian Federation; 2 Center for Theoretical Problems of Physicochemical Pharmacology, Moscow, Russia; 3 Faculty of Physics, Moscow State University, Moscow, Russia

**Keywords:** *SLFN14*, Inherited thrombocytopenia, macrothrombocytopenia, Platelet dysfunction, Bleeding, Platelet function tests

## Abstract

**Background:**

Platelet-type bleeding disorder 20 (BDPLT20), as known as SLFN14-related thrombocytopenia, is a rare inherited thrombocytopenia (IT). Previously, only 5 heterozygous missense mutations in the *SLFN14* gene have been reported.

**Methods:**

A comprehensive clinical and laboratory examination of a 17-year-old female patient with macrothrombocytopenia and severe mucocutaneous bleeding was performed. Examination was carried out using standardized questionnaires to assess bleeding, high-throughput sequencing (Next Generation Sequencing), optical and fluorescence microscopy, flow cytometry with activation and analysis of intracellular calcium signaling of platelets, light transmission aggregometry and thrombus growth in the flow chamber.

**Results:**

Analysis of the patient’s genotype revealed a previously undescribed c.655 A > G (p.K219E) variant in the hotspot of the *SLFN14* gene. Immunofluorescence and brightfield examination of platelets in the smear showed heterogeneity in cells size, including giant forms over 10 μm (normal size 1–5) in diameter, with vacuolization and diffuse distribution of β_1_-tubulin and CD63. Activated platelets showed impaired contraction and shedding/internalization of GPIb. GP IIb/IIIa clustering was increased at rest and attenuated upon activation. Intracellular signalling study revealed impaired calcium mobilization upon TRAP 35.97 nM (reference range 180 ± 44) and CRP-XL 10.08 nM (56 ± 30) stimulation. Aggregation with ADP, collagen, TRAP, arachidonic acid and epinephrine was impaired in light transmission aggregometry; agglutination with ristocetin persisted. In the flow chamber with a shear rate of 400 s^-1^ platelet adhesion to collagen and clot growth were impaired.

**Conclusion:**

The revealed disorders of phenotype, cytoskeleton and intracellular signaling explain the nature of *SLFN14* platelet dysfunction and the patient’s severe hemorrhagic syndrome.

**Supplementary Information:**

The online version contains supplementary material available at 10.1186/s13023-023-02675-9.

## Introduction

The *SLFN14* on chromosome 17q12 is a member of the Schlafen gene family. The SLFN14 protein exhibits supervisory endoribonuclease activity against aberrant RNA and is involved in ribosome degradation during platelet maturation [1, 2]. Platelet-type bleeding disorder (BDPLT20, OMIM #616,913) caused by autosomal dominant mutations in the *SLFN14* gene is classified as inherited thrombocytopenia (IT).

Five heterozygose single nucleotide substitutions resulting in 4 different changes in amino acid sequence have been reported previously: p.K218E, p.K219N, p.V220D, p.R223W [3–6]. They were all concentrated in one hotspot and led to changes in amino acid sequence in the “ATPases associated with diverse cellular activities” (AAA) protein domain.

Patients had macrothrombocytopenia 68–140 × 10^9^/L and decreased level of the SLFN14 protein by 65–80% [3, 4]. Lumiaggregometry revealed impaired aggregation with adenosine diphosphate (ADP), collagen and protease-activated receptor-1-activating peptide (PAR1-AP), as well as reduced adenosine triphosphate (ATP) secretion from dense granules (DG) [3]. Examination of peripheral blood megakaryocyte progenitors showed a decrease in the number of mature megakaryocytes and a proplatelet elongation defect [4].

Here we present a new case of BDPLT20 in an adolescent female patient and detailed results of comprehensive platelet function testing. The findings are relevant for clinicians who have difficulty in identifying SLFN14-related thrombocytopenia (RT) in the differential diagnosis from immune thrombocytopenia and various ITs.

## Methods

### Blood smears

Blood smears were prepared by the conventional Romanowsky–Giemsa technique. The smear from a healthy 27-year-old female volunteer was used as a control sample.

### Genetic research

Molecular genetic study was carried out by the method of high-throughput sequencing (Next Generation Sequencing, NGS) using a targeted panel of 162 genes for the diagnosis of hereditary bleeding disorders (Supplemental list). Genomic DNA was isolated from whole blood using Wizard® Genomic DNA Purification Kit, Promega (USA). The DNA libraries were prepared using the method of selective hybridization enrichment with a custom panel of probes manufactured by the Roche (Switzerland) according to NimbleGen «SeqCap EZ» protocol. Sequencing was performed on the NextSeq Illumina platform (USA) using paired-end reads up to 120 nucleotides long. The average reading depth in the samples was at least 100×, the coverage of the target region was 99% with a reading depth of at least 10×. Bioinformatics processing was carried out using our own automated algorithm that meets international standards. The clinical relevance of the found variants was determined taking into account the recommendations of the American College of Medical Genetics and Genomics (ACMG) [7]. Confirmation of the genetic variants found by the NGS method, as well as a genetic study of the patient’s mother, was performed by direct Sanger sequencing on a Genetic Analyzer 3500XL capillary sequencer (Applied Biosystems).

### Immunofluorescence microscopy

A previously published method with minor modifications was used to prepare and analyze blood smears [8]. Whole blood was collected by vacuum tubes Vacuette with Sodium citrate. Standard air-dried peripheral blood smears were prepared. Then the samples were fixed and permeabilized with ice-cold acetone for 2 min. The fixed samples were incubated for 30 min in a humid chamber with 10% goat serum to reduce non-specific antibody binding. Staining with primary antibodies was carried out for 1 h at room temperature in a humid chamber. Staining with secondary antibodies was carried out in the dark for 1 h at room temperature in a humid chamber. After each stage of incubation with antibodies, the samples were washed 3 times for 5 min in Dulbecco’s phosphate-buffered saline (DPBS) pH = 7.2. Prepared samples were analyzed by fluorescent microscope. The expression of antigens was assessed in comparison with a normal control stained in parallel.

### Thromboelastography

Citrated native type thromboelastography (TEG) was performed using a Thromboelastograph Analyzer 5000 and disposable cups (Haemonetics Corporation, Braintree, MA, USA) according to manufacturer’s instructions. The assays were performed using citrated blood samples (340 µl) recalcified with 20 µl of 0.2 M CaCl2. We used four parameters: R (reaction time), K (coagulation time), alpha (angle), MA (maximum amplitude). Manufacturer’s reference values were used.

### Platelet Intracellular Signaling and Functional Parameters

The analysis of intracellular calcium signaling in platelets was carried out in a similar way [9]. Whole blood was taken into hirudin-containing (525 IU/ml) vacuum tubes. The blood was supplemented with 2 µM calcium-sensitive fluorophore Fura-Red and 0.1 IU/mL of apyrase. After incubation for 35 min at 37 °C, the blood was diluted to a concentration of 1000 platelets/ml and left at 37 °C for 35 min. Alexa-488 stained human fibrinogen was then added to the samples at a concentration of 100 µg/ml and the samples were analyzed on a BD FACS Canto II flow cytometer. Samples were activated with ADP, CRP-XL, TRAP-6 (SFLLRN). The principles for converting Fura-Red fluorescence to calcium concentration and fibrinogen fluorescence to binding fraction are given in Martyanov et al.

### Light transmission aggregometry

The study of platelet aggregation function in platelet rich plasm (PRP) was performed on the Biola aggregometer (Biola, Moscow, Russia) in accordance with Recommendations for the Standardization of Light Transmission Aggregometry (LTA): A Consensus of the Working Party from the Platelet Physiology Subcommittee of SSC/ISTH PRP was prepared by centrifuging blood samples at 200× g for 10 min. The volume of plasma PRP for the study was 300 µl. The results were assessed by percentage of light transmission after agonists adding. The agonists used were: epinephrine − 5 µM, arachidonic acid (АА) -100 µM, TRAP-6–32 µM, ristocetin − 1.5 mg and ristocetin - low 0.7 mg (NPO Renam, Russia), adenosine diphosphate (ADP) − 5 µM (Sigma-Aldrich, St Louis, MO, USA), collagen − 0.2 mg (NPO Renam, Russia). Aggregation curves were recorded for 10–20 min. As control, blood samples were collected from three healthy adolescent female volunteers with a median age of 16 years (15–17 years).

### Flow perfusion chambers experiments

Thrombus formation experiments were performed as described by Podoplelova, et al. [10], with minor modifications. Glass coverslips (24 × 24 mm, Heinz Herenz, Hamburg, Germany) were cleaned with potassium dichromate, rinsed with distilled water and dried. The cleaned coverslips were coated with 200 µg/ml fibrillary type 1 collagen (Chronolog, Havertown, PA, USA) in buffer (150 mM NaCl, 2.7 mM KCl, 1 mM MgCl2, 0.4 mM NaH2PO4, 20 mM HEPES, 5 mM glucose, 0.5% BSA, pH 7.4) for 40 min in a humid chamber at room temperature, rinsed with distilled water and then assembled as part of the parallel-platelet flow chamber. The chambers were passivated with 4% BSA buffer for 20 min prior to blood perfusion. Blood supplemented with 3,3′- Dihexyloxacarbocyanine iodide (DiOC6) and recalcified with 12 mM of CaCl_2_ was perfused at a flow rate corresponding to shear rate of 400 s^-1^. Epifluorescent images of platelet aggregates were acquired with an Axio Observer Z1 microscope (Carl Zeiss, Jena, Germany) equipped with a 100× microscopic objective. Images were analyzed with ImageJ software. The normal control was assessed in parallel with patient.

Routine coagulation assays were performed as describe earlier [11, 12].

Flow cytometry (FC) study of platelets phenotype were performed out as described previously [13–15]. The control group for a flow cytometry of platelets included six healthy adolescent volunteers, aged 16 to 19 years, with a median of 17 years, including four girls and two boys.

## Results

A 17-year-old female patient was referred to our Pediatric Medical Center with thrombocytopenia and life-long history of severe bleeding. She presented at birth and suffered from severe cutaneous bleeding syndrome, nosebleeds, bleeding from minor wounds, after primary teeth replacement and menorrhagia in further life. Bleeding episodes often required emergency medical care and multiple blood transfusion. She had at least two hemarthroses after minor trauma. Her bleeding scores according to both Pediatric Bleeding Questionnaire and ISTH Bleeding Assessment Tool were 20 scores. Severe bleeding history did not correspond to moderate thrombocytopenia (platelets 170 × 10^9^/L at birth, and 60–90 × 10^9^/L further in life). However, symptoms significantly alleviated after the age of 15 years despite of worsening of thrombocytopenia (30–50 × 10^9^/L). At the age of 17 the patient had only easy bruising and heavy menstrual bleeding that required administration of oral tranexamic acid. Clotting tests did not reveal any abnormalities (Supplemental Table 1).

Patient’s mother did not have any bleeding complaints. Patient’s father suffered from severe nosebleed episodes required hospitalization in his childhood and adolescence. He reported easy bruising and, probably, hemarthrosis of knee-joint and had mild thrombocytopenia (100–130 × 10^9^/L). However, his symptoms also clearly alleviated after the age of 20–25 years.

The patient and her mother gave written informed consent for genetic testing. Patient’s father didn’t oppose his daughter investigation but refused his own testing and this circumstance was a limitation of the study.

**Fig. 1:**
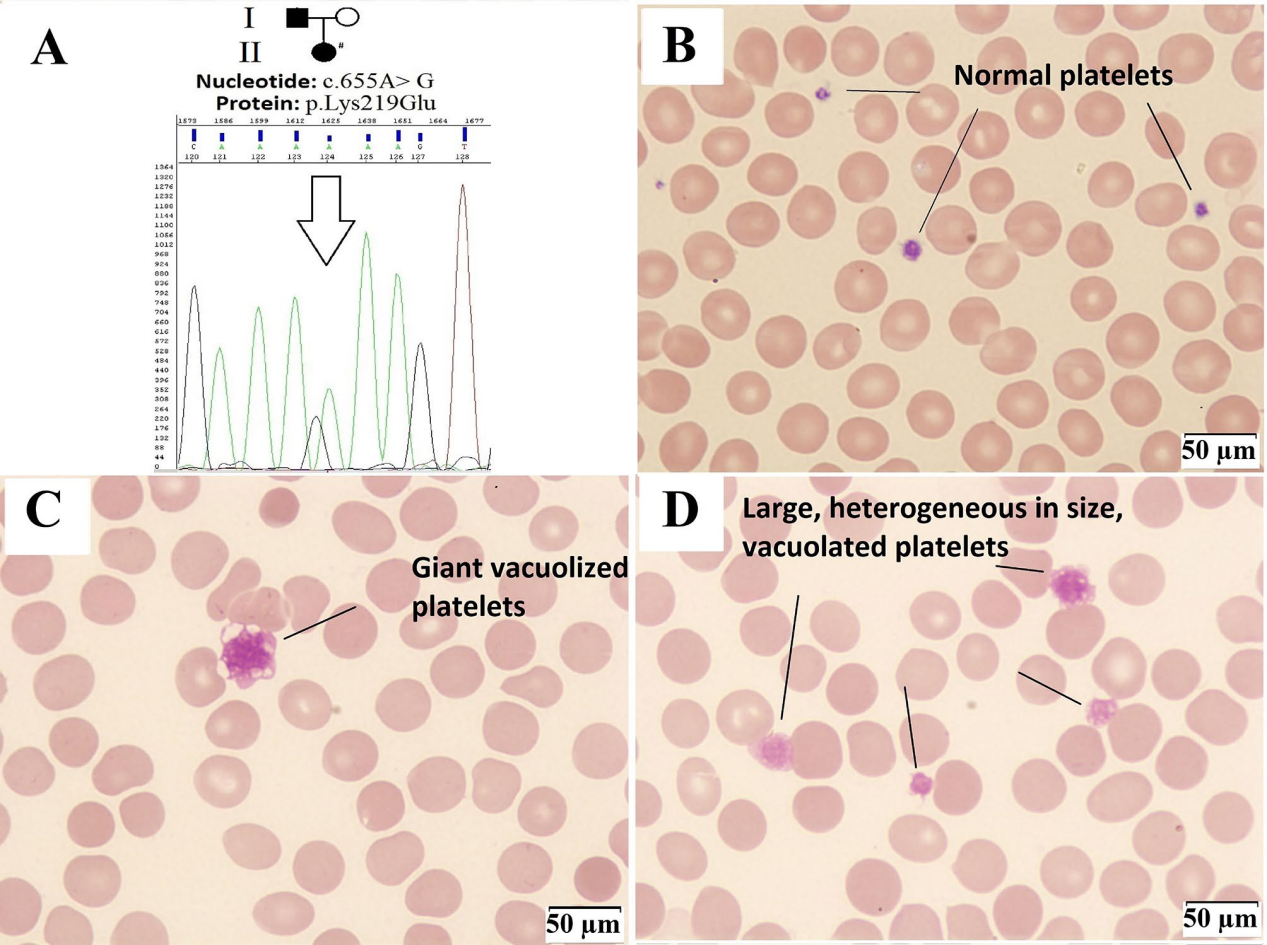
*SLFN14* mutation and optical microscopy of platelets (×1000 magnification). Panel **A** shows affected persons (shaded). The # sign marks the patient, and the arrow indicates the change in nucleotides in the chromatogram. Panel **B** shows a blood smear from a healthy volunteer with normal platelets. Panels **C** and **D** show the patient’s platelets heterogeneous in size with large vacuolar inclusions.

We identified a heterozygous single nucleotide substitution (c.655 A > G) in the *SLFN14* gene (NM_001129820), resulting in the amino acid substitution at position 219 (p.K219E) (Fig. 1A). This genetic variant was not registered in the Human Allelic Variant Database (gnomAD). *In silico* pathogenicity predictors gave conflicting readings for this substitution SIFT: 0.051, tolerated; Polyphen2 HDIV-version: 0.999, damaging; Polyphen2 HVAR-version: 0.996, damaging; PROVEAN: -0.38, neutral, MutationTaster: 0.999, pathogenic; MutationAssessor: 2.435, medium damaging; CADD: 22.9, likely pathogenic.

Two other substitutions, c.657 A > C and c.657 A > T, at the same codon resulting in another amino acid substitution (p.K219N) have been described as pathogenic in families with autosomal dominant platelet disease [3, 5]. Moreover, all previously described pathogenic variants in the *SLFN14* affect codons 218–223, а conserved sequence encoding the AAA domain of the SLFN14 protein [6]. Thus, these codons represent a “hotspot” of mutations. Basing on these arguments, we interpreted the substitution c.655 A > G as a likely pathogenic variant. The substitution was not found in the healthy patient’s mother, and the father’s biomaterial was not available for testing.

Moderate thrombocytopenia (40 × 10^9^/L), with increased fraction of immature platelets (9.7%), 1.5 times higher than the normal values for the patient’s age [16, 17], was found in patient. Their elevation may indicate accelerated platelet clearance, as has been described in immune thrombocytopenia and thrombotic thrombocytopenic purpura [16, 18, 19].

Peripheral blood smear demonstrated platelet heterogeneity in size and granularity (Table 1). Abnormal irregularity of contours, vacuolization in some platelets, and giant forms with a maximum diameter of more than 10 μm (Fig. 1C, D) were observed in comparison with the control (Fig. 1B). Light microscopy did not reveal any abnormalities in leukocytes and erythrocytes.

**Table 1:** Optical microscopy of platelets and fraction of reticular platelets

	IPF, %	Average diameter, µm		Average area, µm²	Fraction < 2.0 μm, %	Fraction 2.0–4.0 μm, %	Fraction 4.0–5.0 μm, %	Fraction > 5.0 μm, %	Ley, %
Norms	1.4–6.4	1.69–2.63	4.03–4.75		44.67	53.54	1.76	0.13	4-9.85
Patient	**9.7**	**3.47**	**10.13**		**2.3**	**72.41**	**19.54**	**5.75**	**3.85**

IPF – immature platelet fraction; Ley – leukocytes; values in bold are out of the normal range.

**Fig. 2:**
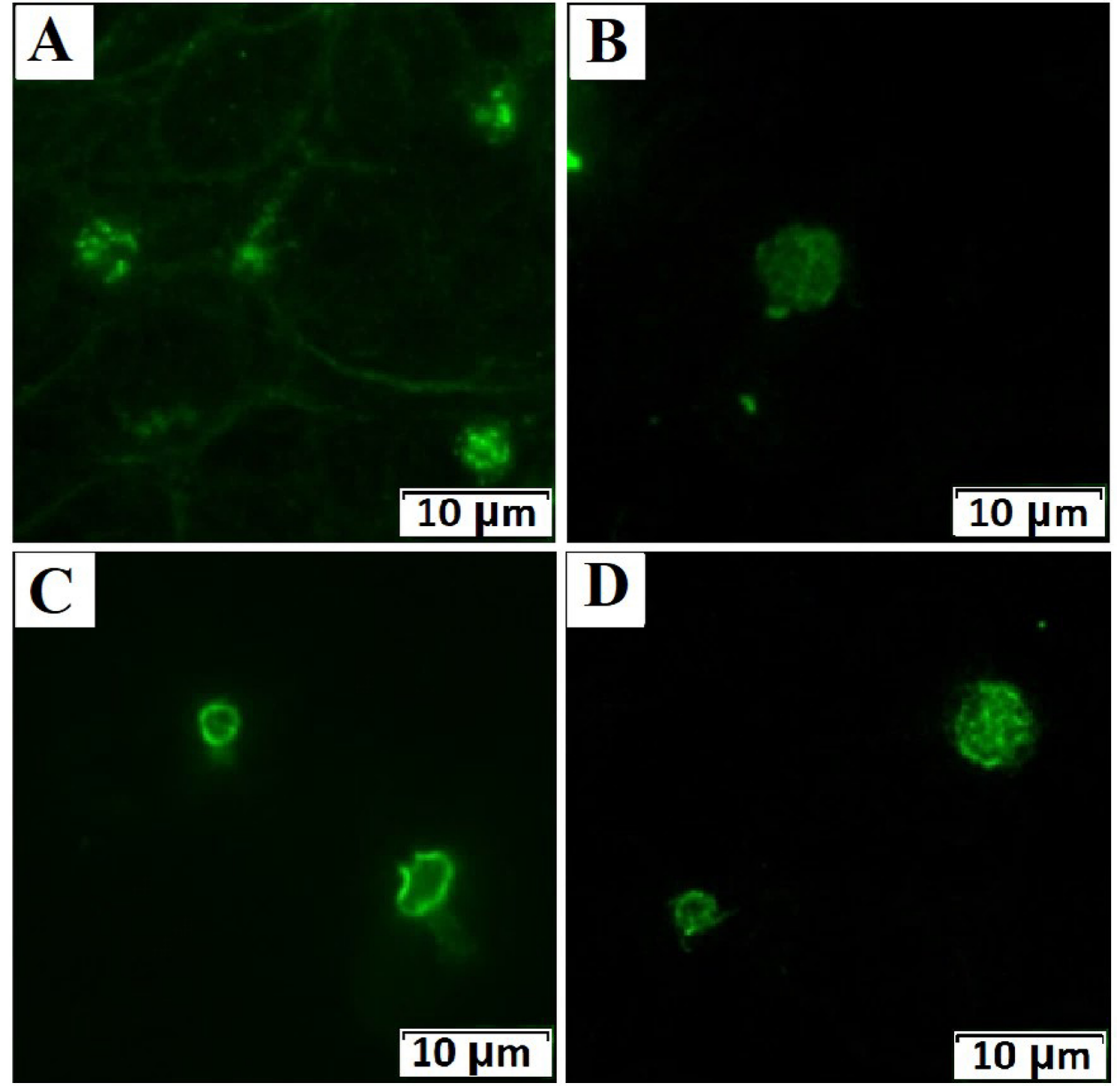
Immunofluorescence microscopy of platelets (×1000 magnification). Panel **A** shows the normal distribution of CD63 dense granules from a healthy volunteer, panel **B** shows a diffuse distribution of CD63 in the patient’s platelets. Panel **C** shows normal expression of β_1_-tubulin, panel **D** shows diffuse distribution of β_1_-tubulin in the patient’s platelets.

Immunofluorescence microscopy of platelets (Supplemental Table 2) showed normal expression of membrane GP Ib/IX and IIb/IIIa. α-Granules markers (P-selectin and von Willebrand factor) were expressed normally as well as a lysosome marker LAMP1. The DG marker LAMP2 was distributed normally, but CD63 was diffusely distributed (Fig. 2B), in comparison with the control (Fig. 2A). α-Tubulin had a normal distribution in the form of rings on the periphery of platelets. β_1_-Tubulin in enlarged platelets was predominantly diffusely distributed (Fig. 2D), in comparison with the control (Fig. 2C).

FC also revealed platelet heterogeneity in size and granularity, assessed by Forward scatter (FSC) and Side scatter (SSC) and showed usefulness of light scattering for identification of morphological alterations (Supplemental Fig. 1). There was no normal decrease in platelet size and granularity upon activation (Table 2). For these reasons the relative values at rest/after activation were calculated to exclude a false interpretation of absolute values of changes upon activation of markers.

Despite the increased size of platelets at rest, the expression of GPIb, GPIIIa and P-selectin in patients’ platelets at rest was comparable to control group (CG) values. After activation, there was an increase in GPIb expression and ratio GPIb at rest / after activation was decreased, indicating a weakening of the shedding / internalization of this subunit. GPIIIa expression did not differ from CG, but PAC-1 binding was increased at rest and decreased after activation. The activation ratio of GP IIb/IIIa (according to PAC-1) was also reduced more than 3 times. In contrast, mepacrine-loaded DG fluorescence was increased in resting platelets, consistent with increased cell size. The DG release ratio did not differ from CG, demonstrating the preservation of the DG release function.

**Table 2:** Flow cytometry of platelets with activation

Platelet parameters	Control range	Patient	Units
Platelet count	229 (219–376)*	**40**	×10^9^/L
FSC (platelet size)			
At rest	107 (81–121)	**131**	%
Activated	69 (54–79)	**100**	%
FSC ratio at rest / after activation	1.53 (1.37–2.09)	**1.31**	Ratio
SSC (platelet granularity)			
At rest	84.6 (70-93.4)	**118**	%
Activated	84.2 (57.5–66.9)	**121**	%
SSC ratio at rest / after activation	1.19 (1.11–1.58)	**0.98**	Ratio
GP Ib (CD42b antigen)			
At rest	99 (68–118)	107	%
Activated	57 (46–71)	**79**	%
CD42b ratio at rest/after activation	1.71 (1.43–2.39)	**1.35**	Ratio
GP IIIa (CD61 antigen)			
At rest	98 (91–113)	104	%
Activated	261 (211–352)	352	%
CD61 ratio after activation / at rest	2.58 (2.28–3.45)	3.38	Ratio
PAC-1 binding (activated GP IIb/IIIa)			
At rest	3.31 (3.02–4.1)	**6.1**	%
Activated	116 (76–165)	**45**	%
PAC-1 ratio after activation / at rest	36 (21–48)	**7.38**	Ratio
Fluorescence of loaded mepacrine in dense granules			
At rest	85 (73–118)	**156**	%
Activated	22 (19–35)	**57**	%
Dense granules release Index at rest/after activation	4.1 (2.2–4.8)	2.7	Ratio
P-selectin (CD62p antigen)			
At rest	2.35 (2.1-4)	2.8	%
Activated	93 (78–114)	99	%
Release index of α-granules (by CD62p) after activation / at rest	41 (20-47.5)	35.4	Ratio
Fraction of procoagulant PS + platelets (by Annexin V)			
At rest	0.3 (0.05–0.7)	0.32	%
Activated	19 (6.2–37.1)	14.0	%

*median (min-max); values in bold are out of the normal range.

Despite the increased size of platelets, P-selectin at rest and upon activation did not differ from CG, which may be due to a decrease in the count or volume of α-granules in the affected platelets. However, the degree of degranulation of the α-granules was normal, indicating that the release mechanisms were preserved (Table 2).

The fraction of procoagulant phosphatidylserine-positive platelets (PS+) was normal, but the absolute count was 5 × 10^9^/L, compared with 34 (13–104) ×10^9^/L in CG. Thromboelastogram abnormalities (prolonged K 10.8 min (normal range 2–9) and decreased alpha angle 20.7 grad. (normal range 22–58, Supplemental Table 1) may reflect lack of PS + platelets.

**Fig. 3:**
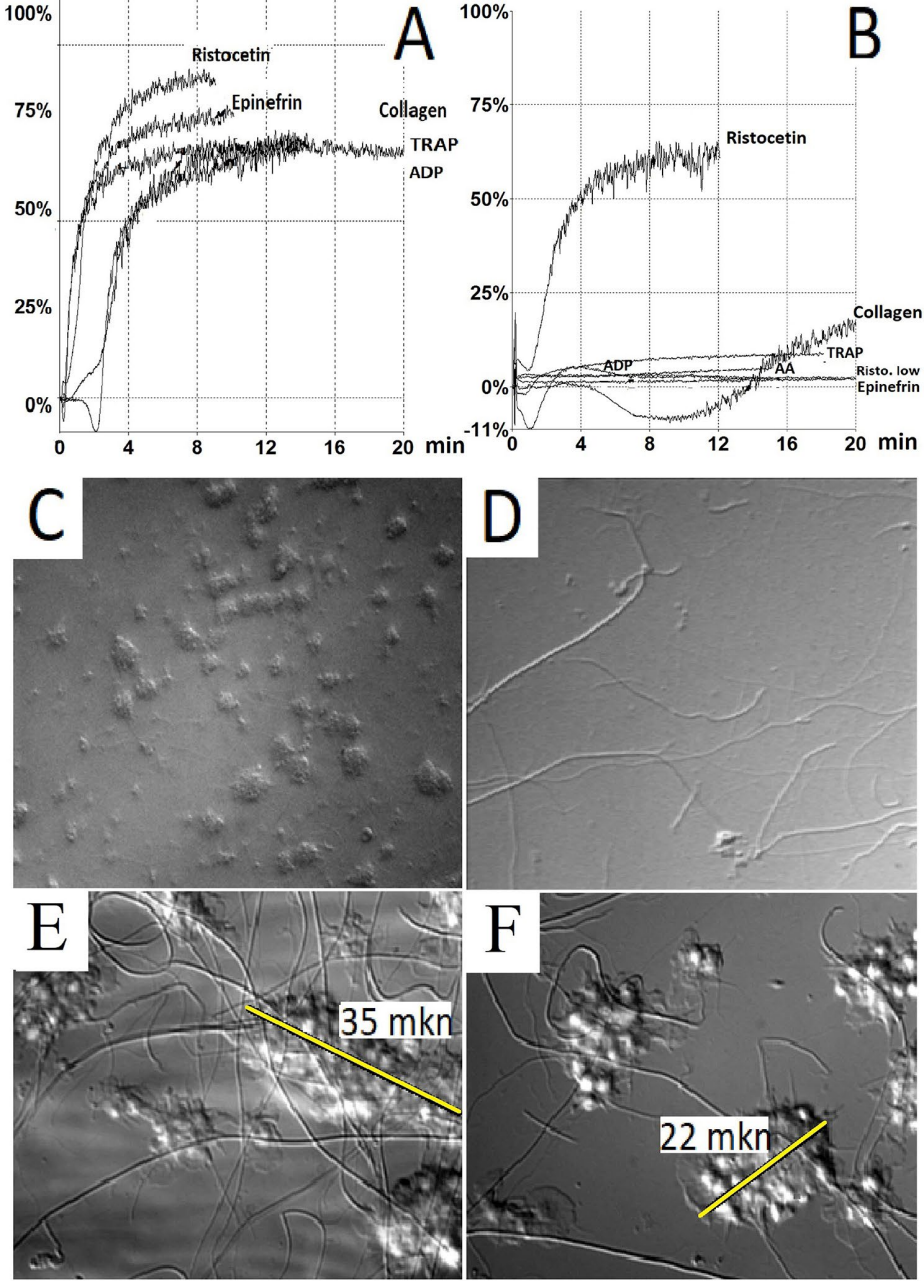
Light transmission aggregometry and growth of thrombi in the flow chamber. Panel **A** shows platelet aggregation curves from a healthy volunteer. Panel **B** shows the patient’s aggregation alteration with all agonists except agglutination/aggregation with ristocetin. The number of thrombus foci was reduced in the patient compared to the control (panels **D** and **C**, respectively, microphotographs at ×20 magnification). The length of the thrombi in the patient was comparable to the control (panels **F** and **E**, respectively, microphotographs at ×100 magnification).

Results of LTA were decreased with epinephrine, arachidonic acid, TRAP-6, ADP and collagen, but were preserved with both ristocetin concentrations (Table 3; Fig. 3B), in comparison with the control (Fig. 3A). The platelet count in platelet-rich plasma was 76 × 10^9^ /L, which is lower than recommended for correct analysis [20].

**Table 3:** Light transmission aggregometry of platelets, %

	PLT in PRP, ×10^9^/L	Epinephrine	AA	TRAP-6	Risto (low)	Risto	ADP	Collagen
Norms	364–570	53–80	42–44	69–84	2.5-9	68–93	56–72	62–79
Patient	**76**	**2**	**5**	**9**	3.5	65	**5 dis.**	**18**

PRP – platelet rich plasm; AA - arachidonic acid; TRAP-6 - thrombin receptor activator peptide-6; Risto – ristocetin; ADP – adenosindiphosphat; Dis. – disaggregation; values in bold are out of the normal range.

Thrombus growth in flow chambers was assessed at a shear rate of 400 s^− 1^, which is typical for the arterial bed, but does not require the involvement of von Willebrand factor in platelet adhesion and aggregation. There was a pronounced decrease in the foci of adhesion of thrombi to the collagen substrate in the fields of view (Fig. 3D), in comparison with the control (Fig. 3C). In the control, the height of the thrombi was 6–14 μm, and the length at the base of the thrombi was 21–35 μm. In the few thrombi of the patient, the height varied from 2 to 5 μm, and the length of the thrombi at the base was 17–35 μm (Fig. 3E, F).

Analysis of intracellular calcium signaling showed that the patient’s resting platelets had a normal calcium concentration. However, the calcium response to platelet activation by ADP was significantly increased, while platelet activation by CRP-XL or TRAP-6 was attenuated. Conversely, fibrinogen binding in response to CRP-XL and TRAP-6 activation was significantly enhanced (Table 4).

**Table 4:** Intracellular platelet signaling

	Calcium-patient, nM	Calcium-norm, nM	Fibrinogen-patient, %	Fibrinogen-norm, %
Preactivation	10 ± 4	19 ± 15	3.3 ± 1.0	3.3 ± 1.3
ADP, 4 µM	**154**	69 ± 32	6.3	8.9 ± 4.7
CRP-XL, 0.5 µg/ml	**10.08**	56 ± 30	**9.1**	2.1 ± 1.8
TRAP-6, 2 µM	**35.97**	180 ± 44	**14.2**	8.8 ± 3.7

These results are inconsistent with decreased PAC-1 binding (Table 2). One of the reasons for this phenomenon may be that PAC-1 binds only to clusters of integrins, while fibrinogen also binds to individual molecules of activated integrins.

## Discussion

We revealed a complex of platelet abnormalities in the reported patient with BDPLT20:

1. An increase in the mean platelet size was combined with signs of impaired platelet contraction upon activation;
2. The distribution of β_1_-tubulin and CD63 in large platelets was diffuse;
3. Intracellular calcium mobilization was impaired and GP IIb/IIIa clustering as well as GP Ib shedding / internalization upon activation were weakened.

S.J. Fletcher et al. previously reported that SLFN14-RT patients had a weakening of aggregation with ADP, collagen and PAR1-AP, but response to arachidonic acid was normal [3]. In observed patient aggregation was impaired with all agonists, except ristocetin.

Earlier reported electron microscopy of platelets in patients with *SLFN14* mutations showed normal α-granules, but there was decreased count of DG. Some patients had a slight increase in platelet size [3]. Our patient also had platelets increased in size, but P-selectin of α-granules was not different from CG, the values of DG were elevated, but the degranulation function remained intact. It is known that the DG marker CD63 is detected in endosomal multivesicular bodies that are precursors of dense and α-granules [21, 22], the maturation impairment of which at the stage of megakaryocytopoiesis [23] can potentially explain vacuolization and diffuse distribution of CD63.

The increased granularity at rest may partly be due to increased cell size. After activation, despite the release of α- and dense granules, granularity was increased and SSC ratio at rest/after activation was reduced, which may also be explained by pathological platelet vacuolization.

The revealed diffuse distribution of β_1_-tubulin in large platelets could cause an alteration of the centralization of the microtubule helix during activation and disrupt the mechanisms of change in the shape, spreading and aggregation of platelets during activating effects [21], which is consistent with the previously described defect in proplatelet elongation [4]. Observed defects could contribute to hypoaggregation, weakening of thrombus growth in the flow, and weakening of platelet size reduction upon activation.

Similar disorders of platelet activation have been previously reported in ANKRD26-RT, which combined a weakening of contraction (according to FSC ratio), shedding/internalization of CD42b and an increase in overall granularity (by SSC) [24]. For thrombocytopenias *RUNX1, ANKRD26, ETV6* and *WAS*, similar complexes of disorders have been described, including the combination of vacuolization, disorders of the cytoskeleton, adhesion and spreading of platelets [22, 25–32].

H.F.G. Heijnen et al. reported that upon activation of platelets through PAR-1 and interaction with fibrinogen, cholesterol rafts of the cytoplasmic membrane are redistributed and accumulated at the tips of filopodia, at the leading edge of spreading cells. This is accompanied by a concentration of c-Src tyrosine kinase and tetraspanin CD63 in these domains, and the destruction of rafts suppresses spreading [33]. In turn, the weakening of platelet activation by collagen can be explained by an alteration of platelet tyrosine phosphorylation due to impaired CD63 distribution and which, as a result, may lead to the observed weakening of platelet spreading [34]. It is also known that binding of CD63 to PI 4-kinase causes the recruitment of this signaling enzyme to specific regions of the platelet membrane, where it influences phosphoinositide-dependent signaling and platelet spreading. It is also suggested that CD63 can modulate GP IIb/IIIa-dependent cytoskeletal reorganization [34] and it is possible that the diffuse distribution of CD63 tetraspanin in the patient’s platelets disrupted the formation of platelet membrane rafts upon activation, which led to impaired clustering of GP IIb/IIIa and weakening of the “out-side in” platelet activation [35].

Prophylactic platelet transfusion to reduce the risk of spontaneous bleeding in patients with thrombocytopenia is recommended at 10 × 10^9^ cells/L or less [36]. This indirectly indicates that the severity of hemorrhagic manifestations in the examined patient does not correspond to the severity of thrombocytopenia, and the identified morphological and functional abnormalities contribute decisively to the development of hemorrhagic symptoms. At the same time, the trend towards symptom relief as the patient matures is partly consistent with previously described observations of patients with minimal manifestations of hemorrhagic syndrome [37].

Thus, we confirm the presence of *SLFN14* platelet abnormalities described in the literature and complement them. The identified laboratory changes fit into the general idea of disorders affecting the cytoskeleton, intracellular architecture and intracellular signaling processes in patients with ITs.

## Electronic supplementary material

Below is the link to the electronic supplementary material.

Supplementary Material 1

## Data Availability

Please contact author for data requests.

## Abbreviations

BDPLT20: Platelet-type bleeding disorder 20
IT: Inherited thrombocytopenia
NGS: Next Generation Sequencing.
SIFT: Sorting intolerant from tolerant
Polyphen2: Polymorphism Phenotyping v2
PROVEAN: Protein Variation Effect Analyzer
CADD: Combined Annotation Dependent Depletion
TRAP: Protease-activated receptor-1-activating peptide
CRP-XL: Collagen related peptide
GP –: Glycoprotein
ADP: Adenosine diphosphate
ATP: Adenosine triphosphate
DG: Dense granules
CG: Control group
FC: Flow cytometry
LTA: Light transmission aggregometry
IPF: Immature platelet fraction
Ley: Leukocytes
LAMP: Lysosomal-associated membrane protein
FSC: Forward scatter
SSC: Side scatter
PS+: Procoagulant phosphatidylserine-positive platelets
PRP: Platelet rich plasm
Risto.: Ristocetin
Dis.: Disaggregation
RT: Related thrombocytopenia
WAS: Wiskott-aldrich syndrome
