# Supplementary material for: Novel SLFN14 mutation associated with macrothrombocytopenia in a patient with severe haemorrhagic syndrome
Source: Orphanet J Rare Dis. 2023 Apr 11;18:74. doi: 10.1186/s13023-023-02675-9 (PMC10091655; doi:10.1186/s13023-023-02675-9)
Supplement: Supplementary file 1 — Supplementary Material 1 [file 13023_2023_2675_MOESM1_ESM.docx]

**Supplementary data**

**The list of genes included in the diagnostic targeted panel for the NGS testing of hereditary bleeding disorders patients.** All exons, adjacent parts of introns, entire 5’UTRs and partly 3’UTRs were included in the target region for SeqCap probes selection (*Roche*, Switzerland).

*ABCA1, ABCD4, ABCG5, ABCG8, ACTB, ACTN1, ACVRL1, ADAMTS13, ANKRD26, ANO6, AP3B1, AP3D1, ARPC1B, BLOC1S3, BLOC1S6, CBS, CD36, CD83, CHST14, COG6, COL1A1, COL1A2, COL3A1, COL5A1, COL5A2, CPB2, CYCS, DIAPH1, DPAGT1, DTNBP1, ENG, EPHB2, ETV6, F10, F11, F12, F13A1, F13B, F2, F2R, F2RL3, F5, F7, F8, F9, FERMT3, FGA, FGB, FGG, FLI1, FLNA, FYB1, GALE, GATA1, GFI1B, GGCX, GNAI3, GNAQ, GNAS, GNE, GP1BA, GP1BB, GP5, GP6, GP9, HABP2, HOXA11, HPS1, HPS3, HPS4, HPS5, HPS6, HRG, IFNA17, IFNAR1, IFNLR1, IKZF1, IL10, ITGA2, ITGA2B, ITGB3, KDSR, KLKB1, KNG1, LMAN1, LMBRD1, LYST, MASTL, MCFD2, MECOM, MGAT2, MLPH, MMAA, MMAB, MMACHC, MMADHC, MMUT, MPI, MPIG6B, MPL, MYH9, MYO5A, MYSM1, NBEA, NBEAL2, NFE2, NSMCE3, ORAI1, P2RX1, P2RY12, PEPD, PLA2G4A, PLAT, PLAU, PLCB2, PLG, PMM2, PRDX1, PRF1, PRKACG, PROC, PROCR, PROS1, PROZ, PTPRJ, RAB27A, RASGRP2, RBM8A, RUNX1, SC5D, SERPINA10, SERPINC1, SERPIND1, SERPINE1, SERPINF2, SLC35A1, SLC35A2, SLC46A1, SLFN14, SMAD2, SMAD4, SRC, SRD5A3, STIM1, STX11, STXBP2, TBXA2R, TBXAS1, TFPI, TFR2, THBD, THPO, TMEM165, TPM4, TRPM7, TUBB1, UNC13D, VIPAS39, VKORC1, VPS33B, VWF, WAS*

**Supplementary table 1** Blood coagulation tests

|  | Clotting test | | | | | Thromboelastogram | | | |
| --- | --- | --- | --- | --- | --- | --- | --- | --- | --- |
|  | PI by Quick, % | APTT, sec. | TT, sec. | Fg, g/L | vWF act., % | R, min | K, min | Angle, grad. | MA, mm |
| Norms | 70-120 | 25,1-36,5 | 15,8-24,9 | 2-3,93 | 60,8-239 | 9-27 | 2-9 | 22-58 | 44-64 |
| Patient | 89 | 32,1 | 23,4 | 2,06 | 80,8 | 15,5 | **10,8** | **20,7** | **40,6** |

PI – prothrombin index; APTT – Activated Partial Thromboplastin Time; TT – thrombin time; Fg – fibrinogen; vWf – von Willebrand factor. R-time; K-time; Alpha angle (α); MA - maximum amplitude; values in bold are out of the normal range.

**Supplementary table 2** Immunofluorescence microscopy of platelets

| Antibody | Healthy volunteer | Patient | Interpretation |
| --- | --- | --- | --- |
| GP ⅠbⅨ | 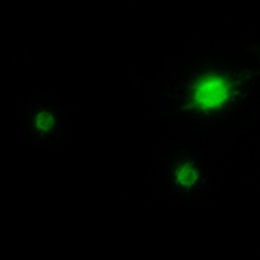 | 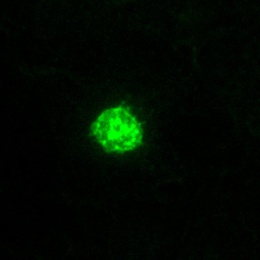 | Normal expression, normal distribution |
| GP ⅡbⅢa | **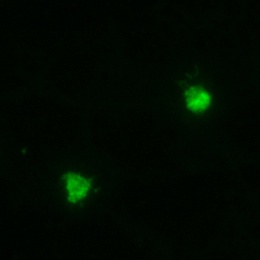** | **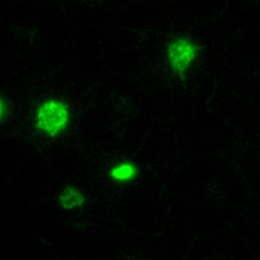** | Normal expression, normal distribution |
| Myosin (platelets) | **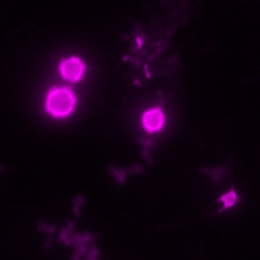** | **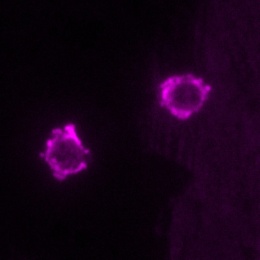** | Normal expression, normal distribution |
| Myosin (granulocytes) | **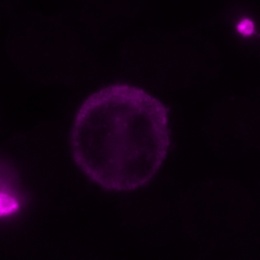** | **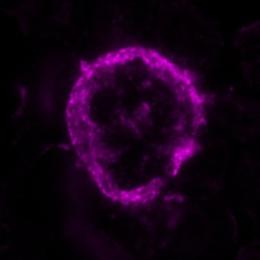** | Normal expression without inclusion bodies |
| Lamp 1 | **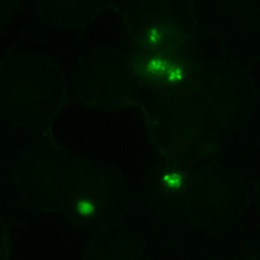** | **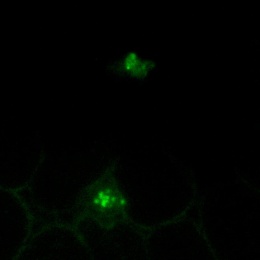** | Normal expression, normal distribution |
| Lamp 2 | **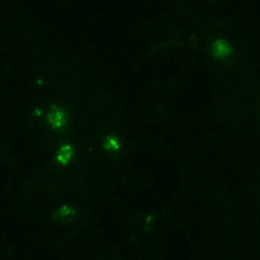** | **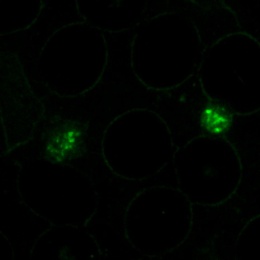** | Normal expression, normal distribution |
| CD 63 | **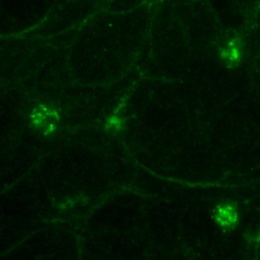** | **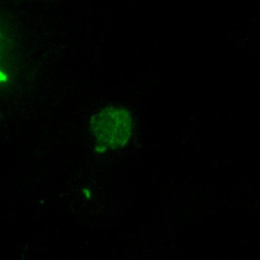** | **Normal expression, diffuse distribution** |
| P – selectin | **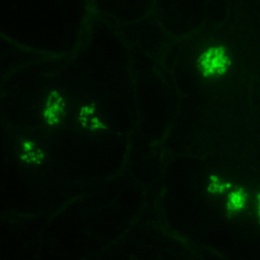** | **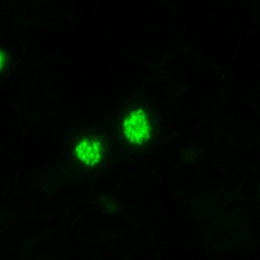** | Normal expression, normal distribution |
| vWF | **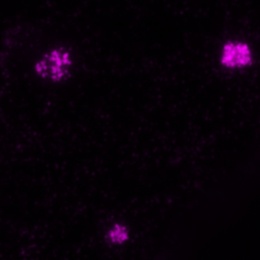** | **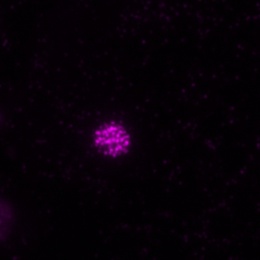** | Normal expression, normal distribution |
| β1 – tubulin | **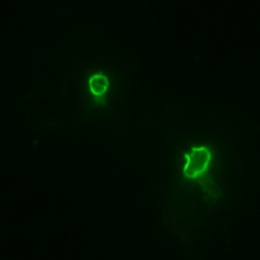** | **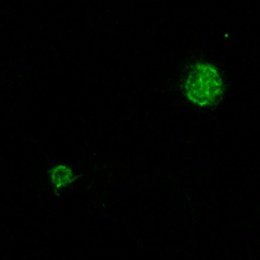** | **Normal expression, some platelets have normal distribution in the form of peripheral rings, but platelets with increased size have diffuse distribution** |
| α – tubulin | **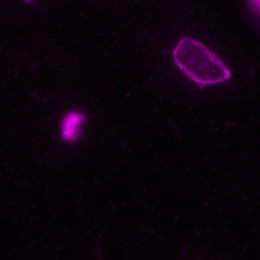** | **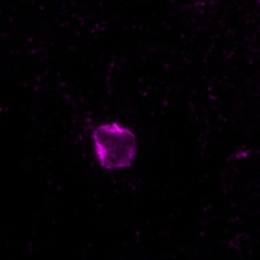** | Normal expression, normal distribution in the form of peripheral rings |

**Supplementary figure 1.**


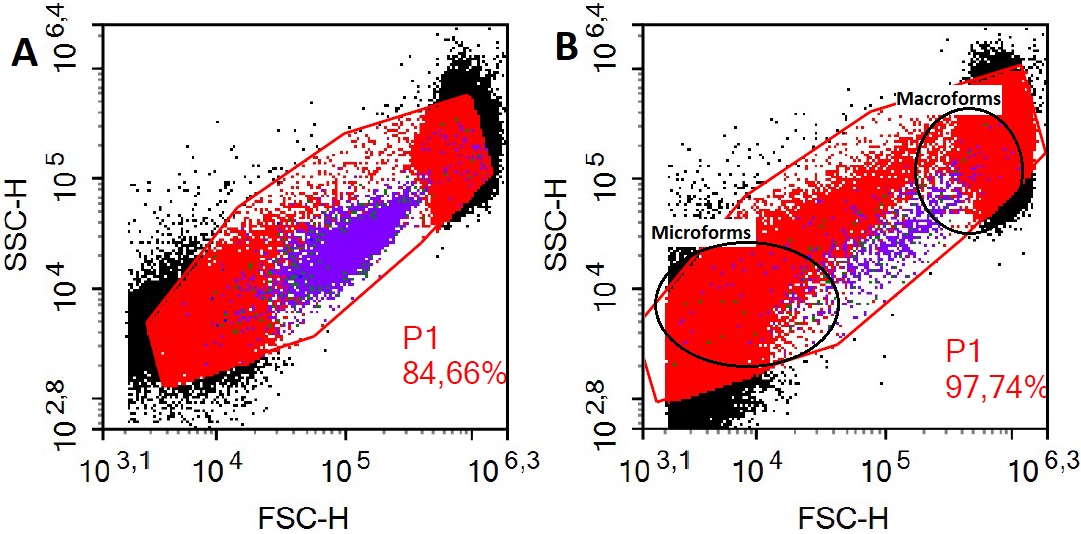


**Supplementary Figure 1** Characteristics of platelets with light scattering. Panel **A** shows the normal distribution of platelet size and granularity in a healthy volunteer (colored in purple). Panel **B** demonstrates the high morphological heterogeneity of the patient's platelets.
